# *Rickettsia africae* in *Amblyomma variegatum* Ticks, Uganda and Nigeria

**DOI:** 10.3201/eid1910.130389

**Published:** 2013-10

**Authors:** Vincenzo Lorusso, Karolina Anna Gruszka, Ayodele Majekodunmi, Augustine Igweh, Susan C. Welburn, Kim Picozzi

**Affiliations:** University of Edinburgh, Edinburgh, Scotland, UK (V. Lorusso, K.A. Gruszka, A. Majekodunmi, S.C. Welburn, K. Picozzi);; Nigerian Institute for Trypanosomiasis Research, Jos, Nigeria (A. Igweh)

**Keywords:** Rickettsia africae, African tick-bite fever, Amblyomma variegatum, Uganda, Nigeria, rickettsia, ticks, vector-borne infections, spotted fever group

**To the Editor:** *Rickettsia africae* is the most widespread spotted fever group (SFG) rickettsia in sub-Saharan Africa, where it causes African tick-bite fever (*1*), an acute, influenza-like syndrome. The number of cases in tourists returning from safari in sub-Saharan Africa is increasing (*1*). In western, central, and eastern sub-Saharan Africa, *R. africae* is carried by *Amblyomma variegatum* (Fabricius, 1794) ticks (*2*); usually associated with cattle, this 3-host tick also can feed on a variety of hosts, including humans (*2*). *R*. *africae* has not been reported in Uganda and rarely reported in Nigeria (*3*,*4*). Our objective was to determine the potential risk for human infection by screening for rickettsial DNA in *A. variegatum* ticks from cattle in Uganda and Nigeria.

In February 2010, ticks were collected from zebu cattle (*Bos indicus*) from 8 villages in the districts of Kaberamaido (Adektar [1°81′ N–33°22′ E], Awimon [1°66′ N–33°04′ E], Kalobo [1°88′ N–33°25′ E], Odidip [1°90′ N–33°30′ E], and Odikara [1°91′ N–33°30′ E], Olilimo [1°75′ N–33°38′ E], and Dokolo (Alela [2°09′ N–33°16′ E], and Angeta [1°87′ N–33°10′ E]) in Uganda and, in June 2010, in 3 villages (Mangar [9°14′ N–8°93′ E], Ruff [9°43′ N–9°10′ E], and Tambes [9°38′ N–9°38′ E]) in the Plateau State in Nigeria (Figure). This convenience sample was obtained as part of other ongoing research projects in both countries. Ticks were preserved in 70% ethanol and identified morphologically to the species level by using taxonomic keys (*5*). Because the anatomic features do not enable an objective assessment of the feeding status of adult male ticks, engorgement level was determined only in female tick specimens and nymphs.

**Figure:**
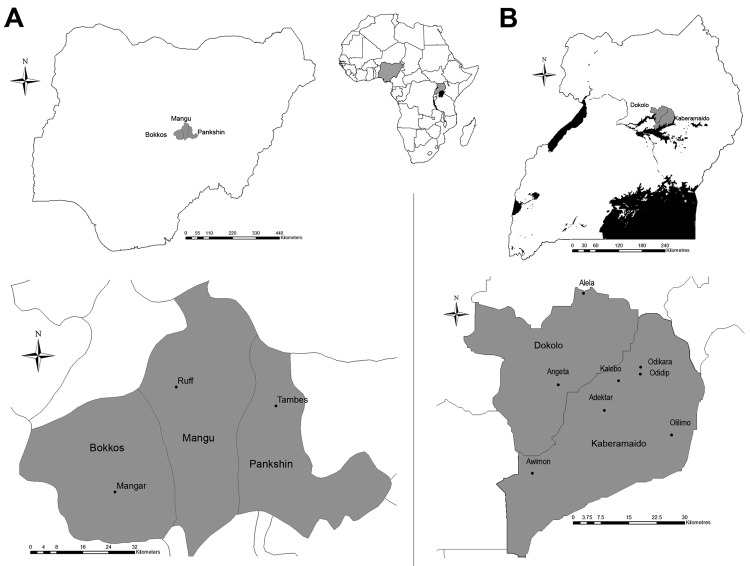
Location of areas studied for *Rickettsia africae* in *Amblyomma variegatum* ticks in Nigeria (A) and Uganda (B), 2010.

After tick identification, DNA was extracted from ticks by using QIAmp DNeasy kits (QIAGEN, Hilden, Germany). Two PCR targets were assessed within each sample; the primer pair Rp.CS.877p and RpCS.1258n was selective for a 396-bp fragment of a highly conserved gene encoding the citrate synthase (*glt*A) shared by all *Rickettsia* spp. (*6*); the Rr190–70p and Rr190–701n primer pair amplified a 629–632-bp fragment of the gene encoding the 190-kD antigenic outer membrane protein A (*omp*A), common to all SFG rickettsiae (*6*,*7*). DNA extracted from 2 *A. variegatum* tick cell lines (AVL/CTVM13 and AVL/CTVM17), previously amplified and sequenced by using primers for *Rickettsia* 16S rRNA, *omp*B, and *sca4* genes revealing >98% similarity with *R. africae* (*8*), was used as a positive control. Negative controls consisted of DNA from 2 male and female laboratory-reared *Rhipicephalus appendiculatus* ticks and distilled water. DNA of positive samples was recovered, and confirmation of amplicon authenticity was obtained through sequence analysis by using nucleotide BLAST (www.ncbi.nlm.nih.gov/BLAST).

A total of 39 ticks were collected in Uganda (32 adult males, 5 females, and 2 nymphs), and 141 were collected in Nigeria (80 males, 59 females, and 2 nymphs); all were identified as *A. variegatum* (Technical Appendix Table). SFG rickettsiae DNA was amplified in 26 (67%) of 39 ticks from Uganda and 88 (62%) of 141 ticks from Nigeria by using the *omp*A gene primers; amplicons of the *glt*A genes were obtained in 16 (41%) of 39 ticks and 84 (60%) of 141 ticks, respectively (Technical Appendix Table). Overall, 81 (45%) of 180 ticks were positive by *glt*A and *omp*A PCRs (Technical Appendix Table). DNA sequences of the 22 *glt*A and *omp*A products from Uganda and the 22 from Nigeria showed 100% similarity with published sequences of *R. africae* (GenBank accession nos. U59733 and RAU43790, respectively). For both countries, ticks positive for *Rickettsia* spp. and SFG rickettsiae DNA were male and female specimens (Technical Appendix Table). Among females, both unengorged and engorged specimens contained DNA from rickettsiae and SFG rickettsiae (Technical Appendix Table).

These findings represent a novelty for Uganda. With reference to Nigeria, our results contrast with the prevalence of 8% recorded in a similarly sized sample (n = 153) of *A. variegatum* ticks collected from cattle in the same part of the country (*3*); this discrepancy might be the result of previous targeting of the rickettsial 16S rDNA gene. In the study reported here, the SFG-specific *omp*A PCR proved to be more sensitive than *glt*A for detecting rickettsiae DNA, as has also been reported in previous work (*9*). Although finding *R. africae* DNA in engorged female and nymphal tick specimens might be attributable to prolonged rickettsemia in cattle (*10*), the presence of *R. africae* in distinctly unengorged female ticks indicates the potential for *A. variegatum* ticks to act as a reservoir of this SFG rickettsia (*2*).

This study extends the known geographic range of *R. africae* in *A. variegatum* ticks in sub-Saharan Africa. The number of potentially infective ticks recorded in Uganda and Nigeria suggests that persons in rural areas of northern Uganda and central Nigeria might be at risk for African tick-bite fever. Awareness of this rickettsiosis should be raised, particularly among persons who handle cattle (e.g., herders and paraveterinary and veterinary personnel). Physicians in these areas, as well as those who care for returning travelers, should consider African tick-bite fever in their differential diagnosis for patients with malaria and influenza-like illnesses.

Technical Appendix Results of PCR screening of *Amblyomma variegatum* ticks.
