# Supplementary material for: Rickettsia africae in Amblyomma variegatum Ticks, Uganda and Nigeria
Source: Emerg Infect Dis. 2013 Oct;19(10):1705–7. doi: 10.3201/eid1910.130389 (PMC3810746; doi:10.3201/eid1910.130389)
Supplement: Technical Appendix — Results of PCR screening of Amblyomma variegatum ticks. [file 13-0389-Techapp-s1.pdf]

Article DOI: http://dx.doi.org/10.3201/eid1910.130389

Rickettsia africae in *Amblyomma variegatum* Ticks, Uganda and Nigeria

Technical Appendix

Technical Appendix Table. Results of PCR screening of *Amblyomma variegatum* ticks, Uganda and Nigeria, 2010*

	No. ticks Identified			No. PCR-positive/total											
				*glt*A				*omp*A				*glt*A and *omp*A			
Study area	M	F	N	Total	M	F	N	Total	M	F	N	Total	M	F	N
Uganda, n = 39	32	5 (4)	2	16/39	12/32	4 (3)/5 (4)	0/2	26/39	24/32	2 (2)/5 (4)	0/2	13/39	11/32	2 (0)/5 (4)	0/2
Nigeria, n = 141	80	59 (28)	2 (1)	84/141	45/80	38 (19)/59 (28)	1 (1)/2 (1)	88/141	44/80	44 (22)/59 (28)	0/2(1)	68/141	32/80	36 (18)/59 (28)	0/2(1)
Total, n = 180	112	64 (32)	4 (1)	100/180	57/112	42 (22)/64 (32)	1 (1)/4 (1)	114/180	68/112	46 (24)/64 (32)	0/4 (1)	81/180	43/112	38 (18)/64 (32)	0/4(1)

*M, male; F, female; N, nymph. Numbers in parentheses indicate engorged female ticks and nymphs.
